# Deformation Twinning Induced High Tensile Ductility of a Gradient Nanograined Cu-Based Alloy

**DOI:** 10.3390/nano11092451

**Published:** 2021-09-20

**Authors:** Junjie Wang, Nairong Tao

**Affiliations:** 1 Shenyang National Laboratory for Materials Science, Institute of Metal Research, Chinese Academy of Sciences, Shenyang 110016, China; jjwang13b@alum.imr.ac.cn; 2 University of Chinese Academy of Sciences, Beijing 100049, China

**Keywords:** nanograins, ductility, twinning, grain coarsening

## Abstract

We investigated the tensile properties of gradient nanograined Cu and CuAl samples prepared by plastic deformation. Tensile tests showed that the gradient nanograined Cu-4.5Al sample exhibits a uniform elongation of ~22% without any cracks, while the uniform elongation of the gradient nanograined Cu sample is only ~18%. Numerous mechanical twinning retards the softening of the nanograins and accommodates a high tensile ductility in the gradient nanograined Cu-4.5Al sample. This work indicates that mechanical twinning is a potential deformation mechanism to achieve high tensile ductility of nanograined materials.

## 1. Introduction

Tensile ductility of nanograined metals is very limited, due to the lack of work hardening and the early occurrence of necking during tension [1,2], which severely impedes the investigation on their tensile properties and industrial applications. The low work hardening is intrinsic for nanograined metals, due to the extremely fine grains. During deformation, dislocations in nanograined metals cannot be accumulated neither in grain interiors nor at grain boundaries [1,3], resulting in very limited tensile ductility. Over the past decades, numerous investigations have been made to understand the tensile deformation of nanograined metals and to raise their tensile ductility [4,5,6,7,8,9,10]. For example, stress-driven grain boundary migration was observed to accommodate the tensile deformation of thin nanograined Al films with an average grain size of 60–90 nm, but strain localization and early necking tended to occur immediately after yielding, due to the absence of work hardening ability, resulting in low tensile ductility [4].

In recent years, several investigations showed that the gradient nanograined copper exhibited a high tensile ductility of ~31% [11], in which strain localization and early necking of nanograins were restricted. Mechanically driven grain boundary migration dominated the plastic deformation of the surface nanograins and contributed to the high uniform elongation. It is noted that nanograin coarsening accompanied by grain boundary migration induced obvious softening. The surface nanograins of gradient structured IF steel can also sustain a high tensile ductility without obvious grain boundary migration observed in the surface nanograins, the high tensile ductility was attributed to the extraordinary strain hardening and back stress strengthening of the gradient structure [12,13]. Obviously, deformation mechanism of nanograins is closely related to the intrinsic nature of materials.

Grain boundary migration might be inhibited in solid solution alloys since solid solution atoms can impede dislocation movement and then pile-up against grain boundary, which reduces the stress concentration and, thus, suppresses grain boundary migration [14]. Solid solution atoms can also reduce grain boundary energy and, thus, reduce grain boundary mobility [15]. As a result, grain boundary migration-induced grain coarsening is inhibited in solid solution alloys. In our previous work on the tensile deformation mechanism of gradient nanograined CuAl alloys [16], less nanograin coarsening was found in the nanograins of the topmost surface layer. In this work, we focus on the mechanical twinning dominated tensile ductility of the gradient nanograined layer without the coarse-grained matrix. The results revealed that the occurrence of mechanical twinning in the gradient nanograined layer raised the work hardening, and thus suppressed softening induced by the grain coarsening at a certain extent, thereby contributing to the uniform ductility in free-standing nanograined materials under tension.

## 2. Materials and Methods

Prior to deformation, as-received Cu and CuAl rods were annealed at 973 K and 1123 K for 2 h, respectively, to obtain homogeneous coarse grains with an average grain size of ~70 µm. Then, the rod samples with a diameter of 10 mm and a length of 20 mm were processed using surface mechanical grinding treatment (SMGT) at ~173 K, the details of which are available in [16,17]. The SMGT treatment was repeated six times to generate a thick and uniform gradient nanograined layer in each sample. No crack was identified in the surface of the treated Cu and CuAl samples. The gradient nanograined layer was extracted from the treated rods to investigate the ductility and tensile deformation mechanism of the nanograins. Microstructures of the longitudinal section of the gradient nanograined Cu and CuAl samples were characterized, using FEI NanoSEM Nova 430 (Hillsboro, OR, USA) field emission gun scanning electron microscope (FEG-SEM) and FEI Tecnai G^2^ F20 transmission electron microscope (TEM) operated at a voltage of 200 kV.

## 3. Results

### 3.1. Microstructures of Gradient Nanograined Cu and CuAl Alloys

The SMGT-treated Cu and CuAl samples show a gradient nanograined layer of ~700 µm thick regardless of Al solute concentration. Formation of the gradient nanograined layer is attributed to the gradient distribution of strains and strain rates during SMGT treatment [16]. The microstructure consists of nanograins in the topmost surface layer of the gradient nanograined Cu (0–20 µm), Cu-2.2Al (0–50 µm) and Cu-4.5Al (0–50 µm) samples. The average grain size of surface nanograins (0–5 µm deep) is ~43 nm, ~35 nm and ~27 nm in the gradient nanograined Cu, Cu-2.2Al and Cu-4.5Al samples, respectively. Some nanograins contain thin deformation twins with both ends terminated at grain boundaries, which was frequently seen in as-deformed nanograins [6,18]. The number fraction of the nanograins embedded with deformation twins is ~3%, ~6% and ~8% in the surface layer (0–5 µm) of the gradient nanograined Cu, Cu-2.2Al and Cu-4.5Al samples, respectively. The microstructure of the subsurface layer is characterized by ultrafine grains in the gradient nanograined Cu/Cu-2.2Al (20–100 µm/50–100 µm) and by nanotwins in the gradient nanograined Cu-4.5Al (50–250 µm). The microstructure is the high density of dislocations in the span from ~100 µm to ~700 µm of the gradient nanograined Cu and Cu-2.2Al samples, while the microstructure is characterized by nanotwins embedded with high density of dislocations in the Cu-4.5Al sample spanning from ~250 µm to ~700 µm. The more detailed microstructure of the gradient nanograined Cu and CuAl samples can be found in the work [16].

### 3.2. Tensile Properties of Gradient Nanograined CuAl Alloys

#### 3.2.1. Tensile Strength and Ductility

**Figure 1:**
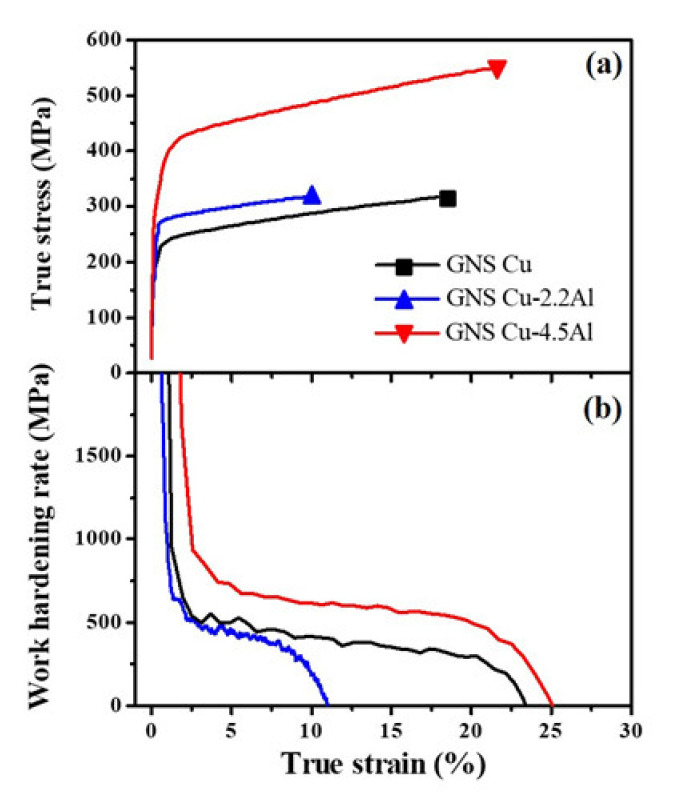
(**a**) True tensile stress-strain and (**b**) work hardening curves of the gradient nanograined Cu, Cu-2.2Al and Cu-4.5Al samples.

Microtensile tests were conducted on dog-bone shaped gradient nanograined Cu and CuAl specimens with a gauge dimension of 3 × 1 × 0.7 mm^3^, using the Instron 5848 microtester machine at a strain rate of 1 × 10^−3^ s^−1^. At least three repetitions were done for each type specimens. The tensile properties here are averaged from more than three tensile data. The tensile results in Figure 1a show that the yield strength of the gradient nanograined Cu, Cu-2.2Al and Cu-4.5Al samples is ~209 MPa, ~256 MPa and ~306 MPa, respectively. Uniform elongation of the gradient nanograined Cu, Cu-2.2Al and Cu-4.5Al samples is ~18%, ~10% and ~22%, respectively. It is noted that uniform elongation of the gradient nanograined Cu-4.5Al sample is the highest (see Figure 1a), and the gradient nanograined Cu-2.2Al sample offers the lowest uniform elongation, which is different from the behavior of their coarse-grained counterparts with uniform elongation increasing with the Al solute concentration.

#### 3.2.2. Work Hardening Rate

Work hardening rate variations against true strain of the gradient nanograined Cu, Cu-2.2Al and Cu-4.5Al samples are shown in Figure 1b. It is seen that all the samples show a steep decrease in the work hardening rate Θ with strain below 2%, corresponding to the elastic–plastic transition. Gradient nanograined Cu-2.2Al sample exhibits the lowest Θ and sharpest Θ drop among the samples tested. With the strain increasing to 11%, Θ of the gradient nanograined Cu-2.2Al decreases to zero. The decreasing rate of Θ in the gradient nanograined Cu-4.5Al sample is lower than that of the gradient nanograined Cu-2.2Al sample and its Θ retains the highest among those samples with strain from 2% to 22%. Gradient nanograined Cu sample exhibits a lower Θ and a comparable decreasing rate of Θ compared with the gradient nanograined Cu-4.5Al sample. The highest Θ in the gradient nanograined Cu-4.5Al sample contributes to the highest ductility, while the lowest Θ in gradient nanograined Cu-2.2Al sample leads to the lowest ductility among the tested samples.

### 3.3. TEM Observations of Gradient Nanograined Cu and CuAl Alloys after Tension

**Figure 2:**
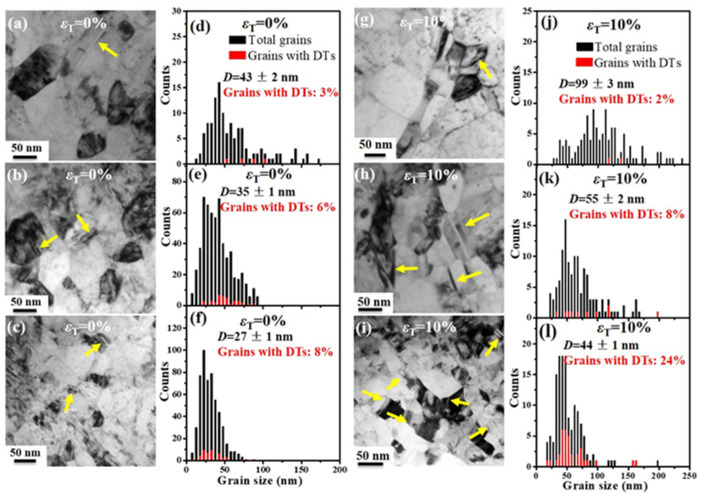
TEM images and corresponding grain size distributions of nanograins (0–5 µm deep) of the gradient nanograined (**a**,**d**) Cu, (**b**,**e**) Cu-2.2 Al, (**c**,**f**) Cu-4.5Al samples before tensile tests, respectively; TEM images and corresponding grain size distributions of nanograins (0–5 µm deep) at 10% tensile strain for the gradient nanograined (**g**,**j**) Cu, (**h**,**k**) Cu-2.2 Al, (**i**,**l**) Cu-4.5Al samples, respectively. The red columns represent the grains embedded with deformation nanotwins (DTs). The yellow arrows indicate the nanograins embedded with DTs.

To investigate the deformation mechanisms of nanograins, TEM observations were performed to characterize the microstructures of the surface nanograins in the gradient nanograined Cu and CuAl samples after tension. As seen in Figure 2, grain coarsening occurs in the nanograins of all the gradient nanograined samples, but the coarsening level becomes less with increasing Al solute concentration in the gradient nanograined Cu and CuAl samples. After a tensile strain of 10%, the average size of the surface nanograins increases to ~99 nm from ~43 nm in the gradient nanograined Cu sample while to ~55 nm from ~35 nm in the gradient nanograined Cu-2.2Al sample and to ~44 nm from ~27 nm in the gradient nanograined Cu-4.5Al sample. Grain coarsening dominates the plastic deformation of the nanograins in the gradient nanograined Cu sample and results in a high tensile ductility of ~18%, which is consistent with our previous study on the gradient nanograined Cu sample, showing a considerable tensile ductility by mechanically driven grain boundary migration and concomitant grain growth [11].

The number fraction of the nanograins embedded with deformation twins decreases by ~1% in the surface layer (0–5 µm) of the gradient nanograined Cu sample after 10% tensile strain (Figure 2d,j). The reason might be that grain coarsening consumes nanotwins since the average grain size increases by ~56 nm after the tensile strain of ~10%. Brons and coworkers [19] also observed remarkable grain coarsening at the expense of twin boundaries in the deformed nanotwinned Cu thin films. The number fraction of the nanograins embedded with deformation twins in the surface layer (0–5 µm deep) of the gradient nanograined Cu-2.2Al and Cu-4.5Al samples increases by ~2% and ~16%, respectively. Obviously, deformation twinning preferentially occurs in the nanograins of the gradient nanograined CuAl samples with an increasing Al solute concentration.

### 3.4. SEM Observations of Surface Nanograins after Tension

**Figure 3:**
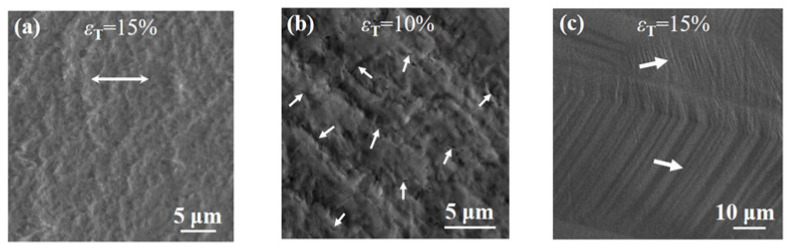
SEM images of surface nanograins (~5 µm deep) in the (**a**) gradient nanograined Cu, (**b**) gradient nanograined Cu-2.2Al and (**c**) gradient nanograined Cu-4.5Al samples with different tensile strains. The inserted white arrow in (**a**) represents the tensile direction. The white arrows inserted in (**b**) indicate the cracks. The white arrows inserted in (**c**) indicate deformation bands.

The gradient nanograined Cu sample deforms uniformly to the strain of ~15% without any crack (Figure 3a), which is consistent with the result in [11]. Nevertheless, small cracks, with a few hundred nanometers in length, are observed in the gradient nanograined Cu-2.2Al sample at a tensile strain of ~5%. With the tensile strain increasing to ~10%, more cracks formed and linked (Figure 3b). Limited deformation twinning and less grain coarsening are inadequate to accommodate the plastic deformation of surface nanograins, resulting in the cracks in the surface nanograins of the gradient nanograined Cu-2.2Al sample at the early stage of plastic deformation. In contrast, there are no cracks in the surface nanograins of the gradient nanograined Cu-4.5Al sample, even at a tensile strain of ~15% (Figure 3c). It is found that there are numerous deformation bands rather than cracks in the surface nanograins. This suggests that the surface nanograins of the gradient nanograined Cu-4.5Al sample exhibit an extraordinary deformation ability.

## 4. Discussion

For the gradient nanograined Cu-4.5Al sample, deformation twinning instead of mechanically driven grain boundary migration is the dominant deformation mode to accommodate the plastic deformation of the nanograins, which contributes to a high tensile ductility of nanograins (~22%). It should be pointed out that the tensile properties in this work were extracted from the specimens with off-standard geometry for tailoring the gradient nanograined metals. The intrinsic properties can be acquired from such off-standard geometry samples since there are abundant grains involved in the gauge section of tensile specimens. A series of tests that we performed also demonstrate that the tensile properties of specimens with varying sizes but an equal geometry ratio are comparable. As seen in Figure 1, the tensile ductility of the gradient nanograined Cu-4.5Al sample exceeds that of the gradient nanograined Cu. This indicates that, besides grain coarsening, deformation twinning is also effective in accommodating the tensile ductility of the nanograins. It is pointed out that only deformation twinning was observed in deformed nanograins in [5,6], but the phenomenon that twinning accommodates a large tensile strain of the nanograins has never been reported. The present work demonstrates that deformation twinning dominates the plastic deformation of the nanograins and accommodates the high tensile ductility of nanograins.

**Figure 4:**
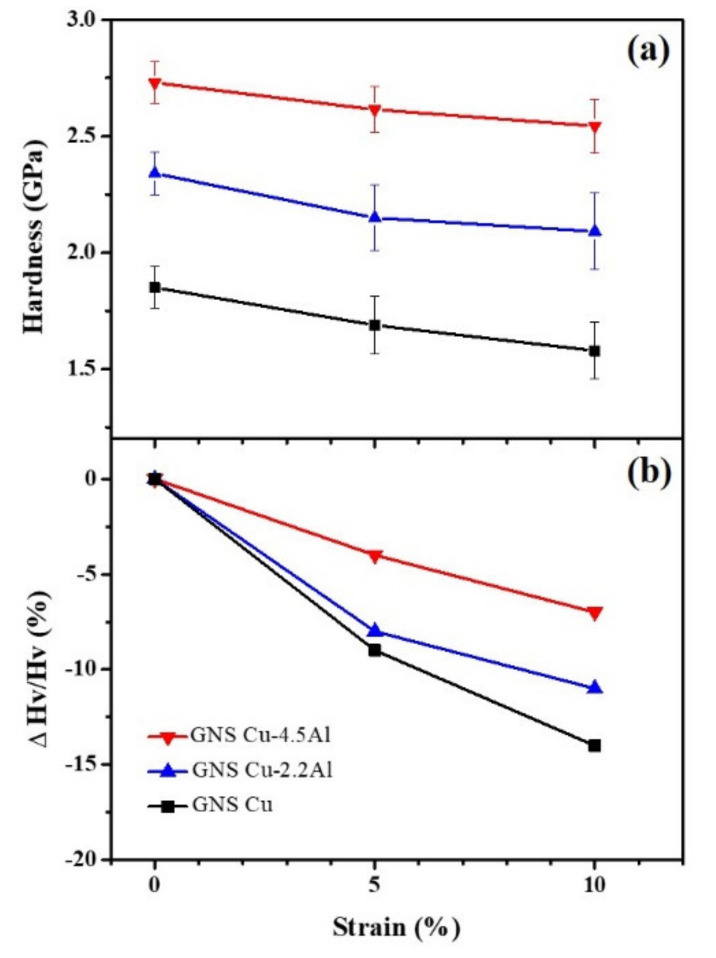
Variations of the (**a**) hardness and (**b**) relative hardness of the surface nanograins (0–5 µm deep) with tensile strain in the gradient nanograined Cu, Cu-2.2 Al and Cu-4.5 Al samples.

Grain coarsening of nanograins generally induces obvious softening during deformation as reflected by the hardness decrease of the nanograins in the gradient nanogrined Cu sample with increased tensile strain [11]. Figure 4 shows the hardness of the surface nanograins at different tensile strains in the gradient nanograined Cu and CuAl samples. All the nanograins of the gradient nanograined samples show softening after tension. At a tensile strain of 10%, the hardness of the surface nanograins in the gradient nanograined Cu sample drops by ~15% (from 1.85 ± 0.09 GPa to 1.58 ± 0.12 GPa), while it drops by ~11% and ~7% for the gradient nanograined Cu-2.2Al and Cu-4.5Al samples, respectively. The softening of surface nanograins is suppressed with increasing the Al solute concentration in the gradient nanograined Cu and CuAl samples, which is attributed to the transition of the deformation mechanism of nanograins from grain coarsening to deformation twinning. As indicated by the TEM observations, the nanograins of the gradient nanograined Cu increase from 43 nm to 99 nm, while they increase from 27 nm to 44 nm with strain up to 10% for the gradient nanograined Cu-4.5Al sample. The number fraction of the surface nanograins embedded with deformation twins reaches ~24% from 8% in the gradient nanograined Cu-4.5Al, while the number fraction decreases to 2% from 3% in the nanograins of the gradient nanograined Cu because of the mechanically driven grain boundary sweeps nanotwins. Obviously, deformation twinning dominates the plastic deformation of the nanograins in the gradient nanograined Cu-4.5Al samples. The results suggest that deformation twinning is an effective way to obtain high strength as well as remarkable work hardening ability of nanostructured materials, which compensates for nanograin coarsening-induced softening to a great extent, and thus, suppresses the softening of the nanograins in the gradient nanograined Cu-4.5Al alloys.

## 5. Conclusions

In this work, a 700 µm-thick gradient nanograined layer was prepared in Cu and CuAl samples via SMGT, respectively. Tensile tests showed the following results:

1. The gradient nanograined Cu-4.5Al sample shows the high work hardening ability and uniform elongation of ~22% without any crack, due to the activation of deformation twinning in surface nanograins.
2. Grain coarsening and mechanical twinning are insufficient for accommodating plastic deformation of surface nanograins in the Cu-2.5Al sample, resulting in the occurrence of surface cracks at a strain of ~10%.
3. More deformation twinning and less grain coarsening suppress the softening of surface nanograins in the gradient nanograined Cu-4.5Al in comparison with the gradient nanograined Cu.
4. This work suggests that deformation twinning is a potential deformation mode to achieve large tensile ductility of nanograined materials.

## Data Availability

Not applicable.
